# Supplementary material for: Observing eruptions of gas-rich compressible magmas from space
Source: Nat Commun. 2016 Dec 21;7:13744. doi: 10.1038/ncomms13744 (PMC5187499; doi:10.1038/ncomms13744)
Supplement: Supplementary Information — Supplementary Figures 1-3, Supplementary Table 1 and Supplementary References. [file ncomms13744-s1.pdf]

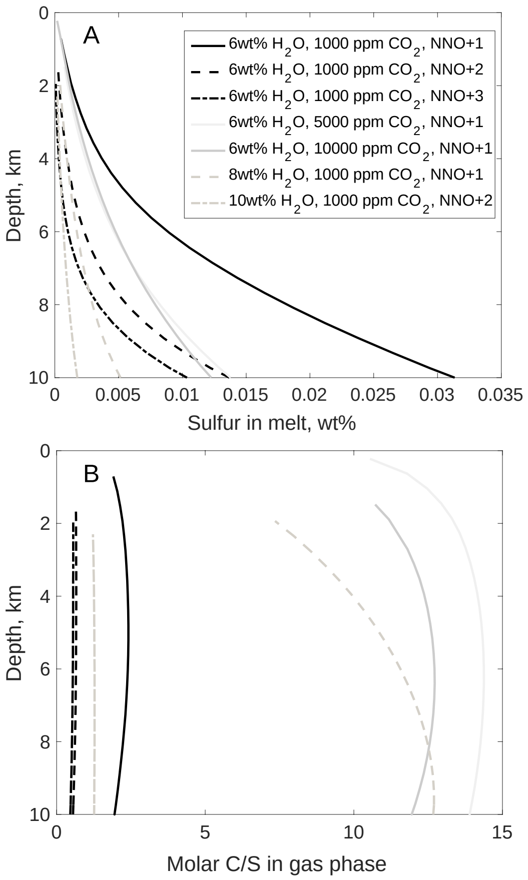

Supplementary Figure 1: **Expected volatile melt concentrations and gas compositions with depth for variable magmatic storage conditions for a metaluminous rhyolite magma**. A: Melt sulfur concentrations with depth for a range of conditions and bulk volatile contents, showing that shallower chambers, or magmas richer in CO_2 will contain more sulfur-poor melts. B: Vapor composition as a function of magma storage depth, showing the C/S ratio of gas coexisting with magma prior to eruption

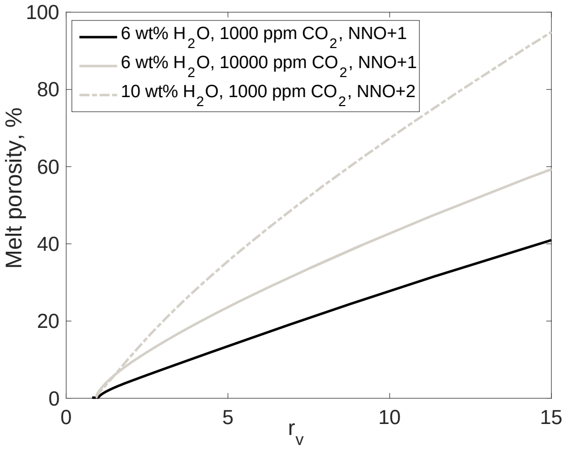

Supplementary figure 2: **Magma porosity as a function of r (proportional to fraction of exsolved gas) and bulk volatile contents.**

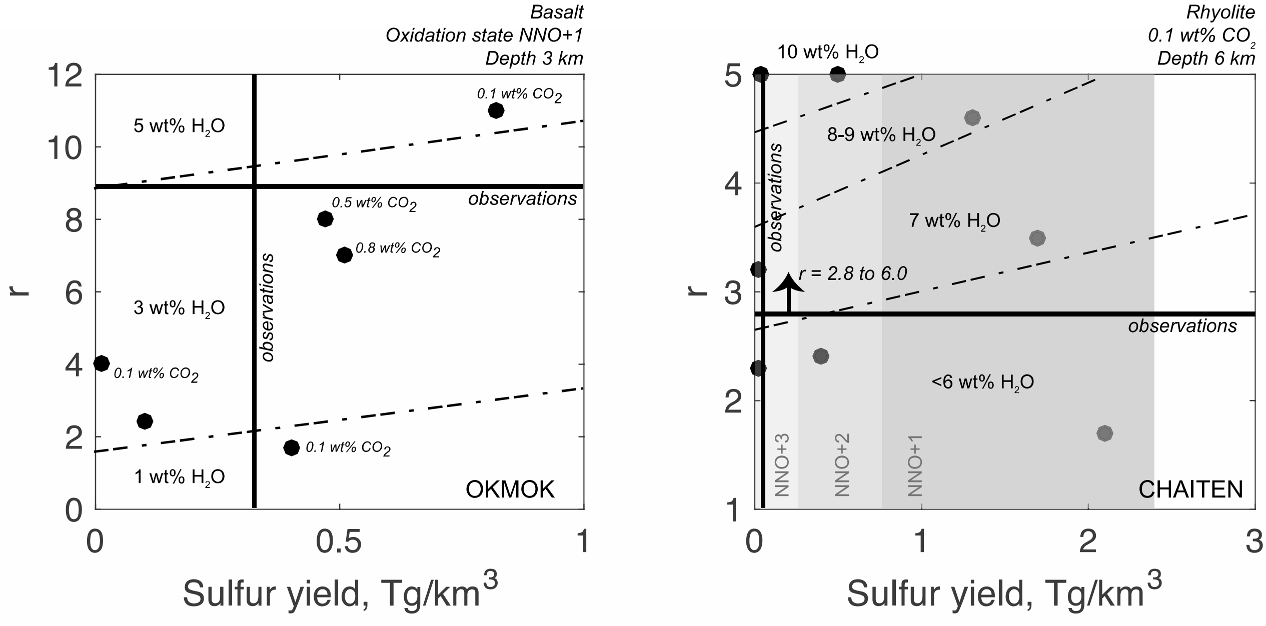

Supplementary figure 3: Analysis of the sulfur yield and extent of ground deformation for two recent volcanic eruptions. Plots show the ratio r plotted against sulfur yield (Tg/km^3). Model runs are shown as black dots and the observations (**Table 1**) are shown as vertical and horizontal heavy black lines. The plot is contoured for melt water content. Left: the 2008 Okmok eruption case, using a basaltic magma composition at NNO+1, stored at a depth of 3 km. Model runs used H_2O contents of 1-5 wt% (marked as regions on the plot) and bulk CO_2 contents of 0.1 to 0.8 wt% (labelled beside each model run). Right: the 2008 Chaiten eruption case, using a rhyolitic melt composition, a bulk CO_2 content of 0.1 wt% and a magma chamber depth of 6 km. Model runs were carried out using a range of f_{O2} (labelled as grey shaded regions) and bulk H_2O contents (regions delineated by black dash-dot lines). The observations yielded an *r* value of 2.8 to 6.0, which is labelled.

Supplementary Table 1: A compilation of space-based ground deformation, gas and erupted volumes for volcanic eruptions. Columns are, from left: volcano name, tectonic setting, date of eruption, magma composition, erupted volume (dense rock equivalent), V_e (DRE, km^3) (minimum and maximum), mass of sulfur emitted in Tg, yield of sulfur in Tg per km^3 magma erupted (minimum and maximum), volume contraction of a model source from ground deformation data ΔV_c (km^3), the ratio r which is equal to $V_e/\Delta V_c$, and the last column contains citations to the papers that yielded these data.

	**Eruption and magma type**			**Erupted volume, km^{3c}**	**Sulfur output (Tg) and yield (Tg/km^3)**		**Syn-eruptive volume change, km^{3e}**	**Source geometry* and depth (km)**	**$r_v = V_e/\Delta V_c$**	**Foot-note**	**Ref.**
**Volcano**	**Setting**[a]	**Date**	**Comp**[b]	V_e	M_S^d	(M_S/V_e)	ΔV_c		r_v		
Chaiten	CA	2/5/08	R	0.7-0.9	0.007	0.008-0.01	0.15-0.25	Ok, 10+/- 2	2.8-6.0		[1,2]
Eyjafjallajokull	OI	14/4/10	B	0.18	0.233	1.3	0.013-0.015	Pe + Ok, 4.0-5.0	12-14		[3,4]
Grimsvotn	OI	21/5/11	B	0.2-0.3	0.15	0.5-0.75	0.027±0.003	Mogi, 1.7	7.4-11		[5-8]
Jebel at Tair	CR	30/9/07	B	0.017±0.008	0.04	1.6-4.4					[9-11]
Kelut	IA	13/2/14	BA	0.2-0.3	0.1	0.3-0.5	-		-		[10,12]
Merapi	IA	29/10/10	A	0.02-0.05	0.15	3-7.5					[10,13,14]
Nabro	CR	13/6/11	BA	0.10	1.825	18	0.07	Mogi, 6.0	1.4		[15-17]
Okmok	IA	12/7/08	BA	0.26	0.075	0.29	0.03	Mogi, 3.0	8.7	1	[18-20]
Puyehue	CA	4/6/11	D	0.25	0.1-0.25	0.4-1.0	0.15	Mogi, 5.0-9.0	1.7	2	[10,21-23]
Soufriere Hills	IA	11/95 to 02/11	A	0.282	1.0	3.5	0.053	Mogi, 10-12	5.3	3	[24-26]
Augustine	CA	29/1/06	A	0.03	0.005	0.17	<0.003	Mogi, 3-5	>10		[10,27,28]
Calbuco	CA	22/4/15	BA	0.11-0.13	0.2	1.5-1.8	-		-		[10,29]
Hekla	OI	26/2/00	BA	0.19	0.0915	0.5	0.04-0.08	Mogi, 14-18	2.4-4.8		[30,31]
Kasatochi	IA	7/8/08	A	0.1	1.1	11	-		-		[10,32]
Nyamuragira	CR	27/11/06	B	0.07	0.5	7.1	-		-		[10,33]
Pdl Fournaise	OI	3/4/07	B	0.13	0.115	0.88	-		-		[34,35]
Redoubt	IA	24/3/09	A	0.08-0.12	0.271-0.308	2.3-3.9	0.028->0.1	Ps, 7.0-15	0.8-4.3	4	[10,36-38]
Sarychev Peak	IA	12/6/09	BA	0.4	0.6	1.5	-		-		[10,39]
Sierra Negra	OI	22/10/05	B	0.15	0.635	4.2	0.08	Mogi + Ok, 2.0	1.9		[10,40]
Anatahan	IA	1/4/05	A	0.05	0.055	1.1					[10,41,42]
El Chichon	CA	1982	D	0.34	7.2	21					[43]
Rabaul	IA	1994	A	0.013	0.42	32					[44]
Fuego	CA	1974	A	0.093	3.1	33					[45]
Ruiz	CA	1985	A	0.029	1	34					[43,46]

Pinatubo	IA	1991	D	4.9	17	3.5					43,47
Mauna Loa	OI	1984	B	0.19	0.22	1.2					48

a IA: island arc; CA: continental arc; OI: ocean island; CR: continental rift.
b B: basalt; BA: basaltic andesite; A: andesite; D: dacite; R: rhyolite.
c: Erupted volumes estimated using various methods, e.g. change in topography, plume height, isopach maps (see citations).
d: Sulfur output measured using satellite-based sensors, e.g. OMI, AIRS (see citations for details).
e: volume change during eruption measured using satellite-based InSAR and/or GPS (see citations).

*Modelled source geometries: Ok: Okada, Pe: penny-shaped crack; Ps: prolate spheroid.

Footnotes
1 Okmok, 2008: erupted volume, modelled source volume and SO_2 emission for first 13 hours, explosive phase only.
2 Puyehue, 2011: Erupted volume for explosive plinian phase only[23]; inferred syn-eruptive volume change of a single spherical source during explosive phase *not* accounting for compressibility – "order of magnitude difference between erupted volume and modelled volume change"[22].
3 Soufriere Hills, 2005-2007: Data for eruptive phase 3 only, 02 Nov 2005 to 01 Apr 2007 deflationary period.
4 Redoubt, 2009: Change in volume of a prolate spheroid with its centroid at $9.17_{15.17}^{6.92}$ km bsl during explosive phase. OMI SO_2 for explosive phase only. Erupted volume for dome + fall +PF deposit volumes during 24 March to 4 April 2009.

Supplementary References

1 Wicks, C., de La Llera, J. C., Lara, L. E. & Lowenstern, J. The role of dyking and fault control in the rapid onset of eruption at Chaitén volcano, Chile. *Nature* **478**, 374-377 (2011).
2 Carn, S. A. *et al.* The unexpected awakening of Chaitén volcano, Chile. *Eos, Transactions American Geophysical Union* **90**, 205-206 (2009).
3 Sigmundsson, F. *et al.* Intrusion triggering of the 2010 Eyjafjallajokull explosive eruption. *Nature* **468**, 426-430 (2010).
4 Carboni, E., Grainger, R., Walker, J., Dudhia, A. & Siddans, R. A new scheme for sulphur dioxide retrieval from IASI measurements: application to the Eyjafjallajökull eruption of April and May 2010. *Atmospheric Chemistry and Physics* **12**, 11417-11434 (2012).
5 Koukouli, M. E. *et al.* Intercomparison of Metop-A SO_2 measurements during the 2010-2011 Icelandic eruptions. *Annals of Geophysics* (2015).
6 Lynch, R. A. Magma Vesiculation and Nature of the Explosive Activity in the 2011 Grímsvötn Eruption. (2015).
7 Sigmarsson, O. *et al.* The sulfur budget of the 2011 Grímsvötn eruption, Iceland. *Geophysical Research Letters* **40**, 6095-6100 (2013).
8 Hreinsdóttir, S. *et al.* Volcanic plume height correlated with magma-pressure change at Grimsvotn Volcano, Iceland. *Nature geoscience* **7**, 214-218 (2014).
9 Clarisse, L. *et al.* Tracking and quantifying volcanic SO 2 with IASI, the September 2007 eruption at Jebel at Tair. *Atmospheric Chemistry and Physics* **8**, 7723-7734 (2008).
10 Carn, S., Clarisse, L. & Prata, A. Multi-decadal satellite measurements of global volcanic degassing. *Journal of Volcanology and Geothermal Research* **311**, 99-134 (2016).
11 Xu, W. & Jónsson, S. The 2007–8 volcanic eruption on Jebel at Tair island (Red Sea) observed by satellite radar and optical images. *Bull Volcanol* **76**, 1-14 (2014).
12 Nakada, S. *et al.* in *AGU Fall Meeting Abstracts.* 06.
13 Costa, F., Andreastuti, S., de Maisonneuve, C. B. & Pallister, J. S. Petrological insights into the storage conditions, and magmatic processes that yielded the centennial 2010 Merapi explosive eruption. *Journal of Volcanology and Geothermal Research* **261**, 209-235 (2013).
14 Saepuloh, A., Urai, M., Aisyah, N., Widiwijayanti, C. & Jousset, P. Interpretation of ground surface changes prior to the 2010 large eruption of Merapi volcano using ALOS/PALSAR, ASTER TIR and gas emission data. *Journal of Volcanology and Geothermal Research* **261**, 130-143 (2013).
15 Hamlyn, J. E. *et al.* Seismicity and subsidence following the 2011 Nabro eruption, Eritrea: Insights into the plumbing system of an off-rift volcano. *Journal of Geophysical Research: Solid Earth* **119**, 8267-8282 (2014).
16 Fromm, M. *et al.* Correcting the record of volcanic stratospheric aerosol impact: Nabro and Sarychev Peak. *Journal of Geophysical Research: Atmospheres* **119** (2014).
17 Goitom, B. *et al.* First recorded eruption of Nabro volcano, Eritrea, 2011. *Bull Volcanol* **77**, 1-21 (2015).
18 Lu, Z. & Dzurisin, D. Ground surface deformation patterns, magma supply, and magma storage at Okmok volcano, Alaska, from InSAR analysis: 2. Coeruptive deflation, July–August 2008. *Journal of Geophysical Research: Solid Earth (1978–2012)* **115** (2010).
19 Larsen, J. F., Śliwiński, M. G., Nye, C., Cameron, C. & Schaefer, J. R. The 2008 eruption of Okmok Volcano, Alaska: Petrological and geochemical constraints on the subsurface magma plumbing system. *Journal of Volcanology and Geothermal Research* **264**, 85-106 (2013).
20 Prata, A. J., Gangale, G., Clarisse, L. & Karagulian, F. Ash and sulfur dioxide in the 2008 eruptions of Okmok and Kasatochi: Insights from high spectral resolution satellite measurements. *Journal of Geophysical Research: Atmospheres* **115** (2010).
21 Castro, J. M. *et al.* Storage and eruption of near-liquidus rhyolite magma at Cordón Caulle, Chile. *Bull Volcanol* **75**, 1-17 (2013).

22 Jay, J. *et al.* Locating magma reservoirs using InSAR and petrology before and during the 2011–2012 Cordón Caulle silicic eruption. *Earth and Planetary Science Letters* **395**, 254-266 (2014).
23 Silva Parejas, C., Lara, L., Bertin, D., Amigo, A. & Orozco, G. in *EGU General Assembly Conference Abstracts.* 9382.
24 Wadge, G., Herd, R., Ryan, G., Calder, E. & Komorowski, J. C. Lava production at Soufrière Hills Volcano, Montserrat: 1995–2009. *Geophysical Research Letters* **37** (2010).
25 Mattioli, G. S. *et al.* Long term surface deformation of Soufrière Hills Volcano, Montserrat from GPS geodesy: Inferences from simple elastic inverse models. *Geophysical Research Letters* **37** (2010).
26 Christopher, T., Edmonds, M., Humphreys, M. & Herd, R. A. Volcanic gas emissions from Soufrière Hills Volcano, Montserrat 1995–2009, with implications for mafic magma supply and degassing. *Geophysical Research Letters* **37** (2010).
27 Coombs, M. L. *et al.* Timing, distribution, and volume of proximal products of the 2006 eruption of Augustine Volcano. Report No. 2330-7102, (US Geological Survey, 2010).
28 Cervelli, P., Fournier, T., Freymueller, J. & Power, J. Ground deformation associated with the precursory unrest and early phases of the January 2006 eruption of Augustine Volcano, Alaska. *Geophysical Research Letters* **33** (2006).
29 Romero, J. *et al.* Eruption dynamics of the 22–23 April 2015 Calbuco Volcano (Southern Chile): Analyses of tephra fall deposits. *Journal of Volcanology and Geothermal Research* **317**, 15-29 (2016).
30 Ofeigsson, B. G., Hooper, A., Sigmundsson, F., Sturkell, E. & Grapenthin, R. Deep magma storage at Hekla volcano, Iceland, revealed by InSAR time series analysis. *Journal of Geophysical Research: Solid Earth* **116** (2011).
31 Höskuldsson, Á. *et al.* The millennium eruption of Hekla in February 2000. *Bull Volcanol* **70**, 169-182 (2007).
32 Waythomas, C. F. *et al.* The 7–8 August 2008 eruption of Kasatochi Volcano, central Aleutian Islands, Alaska. *Journal of Geophysical Research: Solid Earth* **115** (2010).
33 Smets, B., Wauthier, C. & d'Oreye, N. A new map of the lava flow field of Nyamulagira (DR Congo) from satellite imagery. *Journal of African Earth Sciences* **58**, 778-786 (2010).
34 Staudacher, T. *et al.* The April 2007 eruption and the Dolomieu crater collapse, two major events at Piton de la Fournaise (La Réunion Island, Indian Ocean). *Journal of Volcanology and Geothermal Research* **184**, 126-137 (2009).
35 Bhugwant, C., Siéja, B., Bessafi, M., Staudacher, T. & Ecormier, J. Atmospheric sulfur dioxide measurements during the 2005 and 2007 eruptions of the Piton de La Fournaise volcano: Implications for human health and environmental changes. *Journal of Volcanology and Geothermal Research* **184**, 208-224 (2009).
36 Coombs, M. L. *et al.* Andesites of the 2009 eruption of Redoubt Volcano, Alaska. *Journal of Volcanology and Geothermal Research* **259**, 349-372 (2013).
37 Grapenthin, R., Freymueller, J. T. & Kaufman, A. M. Geodetic observations during the 2009 eruption of Redoubt Volcano, Alaska. *Journal of Volcanology and Geothermal Research* **259**, 115-132 (2013).
38 Bull, K. F. & Buurman, H. An overview of the 2009 eruption of Redoubt Volcano, Alaska. *Journal of Volcanology and Geothermal Research* **259**, 2-15 (2013).
39 Rybin, A. *et al.* Satellite and ground observations of the June 2009 eruption of Sarychev Peak volcano, Matua Island, Central Kuriles. *Bull Volcanol* **73**, 1377-1392 (2011).
40 Geist, D. J. *et al.* The 2005 eruption of Sierra Negra volcano, Galápagos, Ecuador. *Bull Volcanol* **70**, 655-673 (2008).
41 Wicks, C., Yarai, H., Lu, Z. & Helz, R. in *AGU Fall Meeting Abstracts.*
42 McCormick, B., Popp, C., Andrews, B. & Cottrell, E. Ten years of satellite observations reveal highly variable sulphur dioxide emissions at Anatahan Volcano, Mariana Islands. *Journal of Geophysical Research: Atmospheres* **120**, 7258-7282 (2015).

43 Wallace, P. Volcanic SO_2 emissions and the abundance and distribution of exsolved gas in magma bodies. *Journal of Volcanology and Geothermal Research* **108**, 85-106 (2001).

44 Roggensack, K., Williams, S. N., Schaefer, S. J. & Parnell Jr, R. A. Volatiles from the 1994 eruptions fo Rabaul: Understanding large caldera systems. *Science* **273**, 490 (1996).

45 Bluth, G. J., Rose, W. I., Sprod, I. E. & Krueger, A. J. Stratospheric loading of sulfur from explosive volcanic eruptions. *The Journal of Geology* **105**, 671-684 (1997).

46 Krueger, A. J., Walter, L. S., Schnetzler, C. C. & Doiron, S. D. TOMS measurement of the sulfur dioxide emitted during the 1985 Nevado del Ruiz eruptions. *Journal of Volcanology and Geothermal Research* **41**, 7-15 (1990).

47 Bluth, G. J., Doiron, S. D., Schnetzler, C. C., Krueger, A. J. & Walter, L. S. Global tracking of the SO2 clouds from the June, 1991 Mount Pinatubo eruptions. *Geophysical Research Letters* **19**, 151-154 (1992).

48 Sharma, K., Blake, S., Self, S. & Krueger, A. SO_2 emissions from basaltic eruptions, and the excess sulfur issue. *Geophysical Research Letters* **31** (2004).
